# Hard Magnetic Properties and the Features of Nanostructure of High-Temperature Sm-Co-Fe-Cu-Zr Magnet with Abnormal Temperature Dependence of Coercivity

**DOI:** 10.3390/nano13131899

**Published:** 2023-06-21

**Authors:** O. A. Golovnia, A. G. Popov, N. V. Mushnikov, A. V. Protasov, K. G. Pradeep, A. V. Ogurtsov, D. V. Taranov, A. M. Tishin

**Affiliations:** 1 M.N. Mikheev Institute of Metal Physics of Ural Branch of RAS, 620108 Ekaterinburg, Sverdlovsk Region, Russia; golovnya@imp.uran.ru (O.A.G.); protasov@imp.uran.ru (A.V.P.); 2 Institute of Natural Sciences and Mathematics, Ural Federal University, 620002 Ekaterinburg, Sverdlovsk Region, Russia; 3 Correlative Microscopy Lab, Department of Metallurgical and Materials Engineering, Indian Institute of Technology Madras, Chennai 600036, India; kgprad@iitm.ac.in; 4 LLC “POZ-Progress”, 624092 Verkhnyaya Pyshma, Sverdlovsk Region, Russia; poz-progress@yandex.ru (A.V.O.); d.v.taranov@yandex.ru (D.V.T.); 5 Faculty of Physics, M.V.Lomonosov Moscow State University, 119991 Moscow, Russia; tishin@amtc.org

**Keywords:** nanocrystalline cellular structure, permanent magnets, Sm_2_Co_17_, SmCo_5_

## Abstract

This paper presents methods and approaches that can be used for production of Sm-Co-Fe-Cu-Zr permanent magnets with working temperatures of up to 550 °C. It is shown that the content of Sm, Cu, and Fe significantly affects the coercivity (*H*_c_) value at high operating temperatures. A decrease in the content of Fe, which replaces Co, and an increase in the content of Sm in Sm-Co-Fe-Cu-Zr alloys lead to a decrease in *H*_c_ value at room temperature, but significantly increase *H*_c_ at temperatures of about 500 °C. Increasing the Cu concentration enhances the *H*_c_ values at all operating temperatures. From analysis of the dependence of temperature coefficients of the coercivity on the concentrations of various constituent elements in this alloy, the optimum chemical composition that qualifies for high-temperature permanent magnet (HTPM) application were determined. 3D atom probe tomography analysis shows that the nanostructure of the HTPM is characterized by the formation of Sm_2_(Co,Fe)_17_ (2:17) cells relatively smaller in size along with the slightly thickened Sm(Co,Cu)_5_ (1:5) boundary phase compared to those of the high-energy permanent magnet compositions. An inhomogeneous distribution of Cu was also noticed in the 1:5 phase. At the boundary between 1:5 and 2:17 phases, an interface with lowered anisotropy constants has developed, which could be the reason for the observed high coercivity values.

## 1. Introduction

Sm-Co-Fe-Cu-Zr permanent magnets (PMs) based on the intermetallic Sm_2_Co_17_ compound were developed in the 1970s. These PMs are characterized by low temperature coefficients of induction and coercivity compared to other rare-earth PMs and excellent corrosion resistance. Such characteristics extend their practical applications up to temperatures of ~300 °C, especially in the growing market of electromobility. The high temperature stability is usually determined by the high Curie temperatures of main phases (727 °C for SmCo_5_ and 920 °C for Sm_2_Co_17_ compounds). The maximum energy product of the Sm(Co_bal_Fe_0.35_Cu_0.06_Zr_0.018_)_7.8_ PM that was achieved in 2015 was (*BH*)_max_ > 35 MGOe, with the coercivity, _J_*H*_c_, as high as 35 kOe [1], putting these materials on par with the Nd-Fe-B PMs. An especially attractive trait is the absence of expensive Terbium in the composition, without the use of which it was not possible to enhance the operating temperatures of Nd-Fe-B PMs above 230 °C. This trend is reflected by pilot batches of Vacomax 278 HR with *BH*_max_ = 282 kJm^3^ (>35 MGOe) tested in 2022. Magnetic hysteresis properties of Sm-Co-Fe-Cu-Zr PMs originate from their nanocrystalline cellular structure [2,3,4], consisting of Sm_2_(Co,Fe)_17_ (2:17) cells separated by interlayers of the Sm(Co,Cu)_5_ (1:5) phase and Zr-rich lamellar phase SmZr_4_Co_13_-H (*P*-6*m*2) or SmZr_2_Co_9_-2H (*P*-6*m*2) structure [5], running along basal planes across cells and cell boundaries. Magnetic hysteresis originates from pinning of domain walls at the 1:5 phase.

The unique nanostructure of the Sm-Co-based PM is formed as a result of long-term multi-stage heat treatment, including solid solution treatment in the range of 1150–1200 °C, isothermal annealing at 800–850 °C, and slow or stepwise cooling down to 400 °C [6]. Interestingly, the cellular nanostructure evolves after the isothermal annealing of the Sm-Co-Fe-Cu-Zr PM in the range of 800–850 °C; however, it does not provide a high-coercivity state. High coercivity _J_*H*_c_ is observed after slow cooling or annealing, with the temperature decreasing stepwise down to 400 °C. The reason for this behavior is related to the redistribution of elements in the cellular structure [7,8,9,10,11,12,13], which was studied in detail using high-resolution transmission electron microscopy [10,13] and atom probe tomography, as reported in [12,14,15,16]. However, in most cases, such studies do not include temperature dependencies of magnetic hysteresis properties. Unfortunately, in the Sm-Co-Fe-Cu-Zr PM, this approach leads to the loss of important information because, depending on the selected composition, various mechanisms of magnetic hysteresis can be observed at different temperatures.

Development of modern technologies requires the expansion of the operating temperature range up to 550 °C for their application in synchronous motors (high torque PM motors), powertrains, and novel high-torque mobility devices such as eVTOL aircraft, etc. For these and other purposes, high-temperature Sm-Co-Fe-Cu-Zr PMs were developed. The starting point of such PMs was the discovery by A.G. Popov et al. [17] of the abnormal temperature dependence of coercivity *H*_c_ with a local maximum at temperatures of 450–550 °C in Sm-Co-Cu-Zr alloys. After the development of sintered PMs with an operating temperature of up to 500 °C [18] in the 1990s [19], Sm-Co-Fe-Cu-Zr PMs were divided into high-energy (HEPM) and high-temperature (HTPM) permanent magnets [20]. Usually, magnets with an operating temperature of 400 °C and above are referred to as the HTPM [21]. However, such division introduces uncertainty, because Sm-Co-Fe-Cu-Zr PMs with operating temperatures of up to 450 °C may not have an abnormal temperature dependence of the coercivity [22,23].

Recently, on the basis of detailed X-ray diffraction, electron microscopy, and magnetic hysteresis studies, it has been established that the absolute value of the negative temperature coefficient of the coercivity β decreases and then becomes positive with an increase in the volume fraction of the Sm(Co,Cu)_5_ (1:5) phase and Cu content in the cellular structure [24]. It was shown that the contents of Sm, Cu, and Fe significantly affect the coercivity at high temperatures [21]. The coercivity of magnets with a high Cu concentration is quite high at room temperature, and the absolute value of the negative coefficient β exceeds 0.15%/°C [22]. Thus, for applications, it is extremely important to identify the physical nature and technological methods [25], thus allowing for the control of the temperature coefficient of coercivity β in order to achieve the required values of _J_*H*_c_ while maintaining the maximum value of remanent induction *B*_r_ at about 1 kG in the operating temperature range of electric drives.

At the same time, it should be noted that various approaches have been developed recently to further improve the properties of these materials. In [26], the correlation between the chemical composition and arrangement of atoms (regions with different Cu content) and the pinning of magnetic domains has been demonstrated via the nanoscale mapping of magnetic domains. This allows for the development of strategies toward the manipulation of atomic scale defects to achieve a desirable *H*_c_ value. There is also an ongoing study on the effect of torsional deformation (leading to the appearance of amorphous phases in SmCo_5_-Cu and SmCo_5_-Fe nanocomposites [27,28,29]) under high pressure on *H*_c_. The effect of high-power pulse currents and laser processing on the formation of bulk and surface nano- and microstructures, respectively, is also studied.

The aim of this work is to understand the physical mechanisms responsible for the dependence of coercivity on temperature and composition. The measurements of magnetic properties of the HTPM with variable Fe content are presented. Based on this analysis, as well as the analysis of literature data devoted to the study of the HTPM with abnormal temperature dependence of coercivity, the dependencies of the temperature coefficient of coercivity β on the content of constituent elements are plotted, and the element concentration range that is most suitable for developing the HTPM is determined. To elucidate the nature of the abnormal temperature dependence of coercivity, the results of atom probe tomography for the sample with the best high-temperature properties are presented.

## 2. Materials and Methods

The Sm(Co_0.888−*x*_Fe*_x_*Cu_0.09_Zr_0.03_)_7_ (*x* = 0; 0.04; 0.08; 0.12) magnets were prepared via a powder metallurgy method. The samples were sintered at temperatures between 1200 to 1215 °C. The solid solution treatment was performed at a temperature of 1200 °C. To achieve the high-coercivity state, the magnets were annealed at 830 °C for 25 h followed by slow cooling to 400 °C at a rate of 0.5 °C/min for 44 h.

The temperature dependencies of magnetization *M*(*T*) and coercivity *H*_c_(*T*) were measured using spherical samples that were 2–3 mm in diameter, using a vibrating-sample magnetometer Lakeshore 7404 (Lake Shore Cryotronics, Inc., Westerville, OH, USA) in magnetic fields of up to 17 kOe. Before measurements, the samples were magnetized in a pulsed field with a strength of up to 70 kOe. X-ray diffraction studies were performed using a multifunctional diffractometer Empyrean (Malvern Panalytical Ltd., Malvern, UK) in Cu Kα radiation.

Atom probe tomography (APT) measurement was carried out for the sample magnet with an Sm(Co_0.76_Fe_0.12_Cu_0.09_Zr_0.03_)_7_ composition. The needle-shaped APT specimens were prepared using FEI Helios G4 UX dual-beam focused ion beam/scanning electron microscope (FIB/SEM) (FEI Company, part of Thermo Fisher Scientific, Hillsboro, OR, USA). APT measurements [30,31,32,33,34] were performed using the CAMECA LEAP 5000 XR system in the laser mode with a pulse frequency of 200 kHz and an applied laser energy of 30 pJ, while the tip was maintained at 60 K. The data reconstruction and analysis were performed with the Integrated Visualization and Analysis Software (IVAS 3.8.10) of CAMECA Instruments Inc (Fitchburg, WI, USA).

## 3. Results and Discussion

### 3.1. Magnetic Measurements

**Figure 1:**
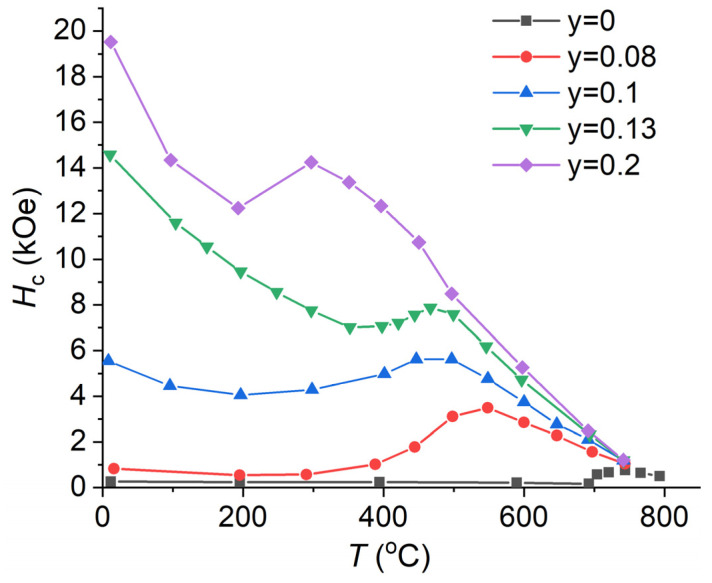
Temperature dependencies of coercivity of the Sm(Co_0.978−*y*_Cu*_y_*Zr_0.022_)_7.3_ magnets, data from [17].

The magnetic nature of the abnormal temperature dependence of coercivity (Figure 1) of the Sm-Co-Fe-Cu-Zr PM is explained in detail in [10,17,35]. With increasing temperature, the domain-wall energy of the 1:5 phase γ_1:5_ decreases more abruptly than that of the 2:17 phase γ_2:17_. This results in a nonmonotonous change in *H*_c_ with local minima and maxima. The initial decrease in *H*_c_ down to its minimum corresponds to the temperature range, in which γ_1:5_ > γ_2:17_ and the domain wall (DW) localizes in the 2:17 phase and is repelled from the 1:5 phase (repulsive pinning). The minimum is formed at the temperature when γ_1:5_ = γ_2:17_. The further increase in temperature corresponds to γ_1:5_ < γ_2:17_, and the DW is attracted by the 1:5 phase (attractive pinning). In this case, coercivity increases (d*H*_c_/d*T* > 0) up to the maximum. Near the maximum *H*_c_, the Curie temperature of the 1:5 phase is reached, and each ferromagnetic 2:17 cell becomes encapsulated by the paramagnetic 1:5 phase. The origin of magnetization reversal changes from the DW pinning to nucleation in the highly anisotropic 2:17 precipitates [17].

The HTPMs with abnormal temperature dependence of *H*_c_ are depleted with Fe and enriched in Sm and Cu. Subsequently, such a composition has a higher volume fraction of the Sm(Co,Cu)_5_ phase. The (*BH*)_max_ of these magnets is approximately 20 MGOe [24,36].

The main characteristics that describe the thermal stability of the PMs are the temperature coefficients of remanence α and intrinsic coercivity β. The former is mainly controlled by the Curie temperature. The β coefficient is calculated as follows [22,37]:

(1) $$\beta (T)=\frac{H_{c}\left(T_{1}\right)-H_{c}\left(T_{0}\right)}{H_{c}\left(T_{0}\right)\left(T_{1}-T_{0}\right)}\;\cdot \;100\%,$$

where *T*_0_ and *T*_1_ are the room temperature and operating temperature, respectively. Thus, similar to coercivity, they are dependent on the magnetic hysteresis and composition of magnets, as well as the intrinsic properties of constituting phases. Additionally, as suggested in [38], β(*T*) can be controlled by the composition and microstructure of the magnet. Thus, to some extent, the unique nanostructure of Sm-Co-Fe-Cu-Zr magnets can be characterized by the β coefficient.

#### 3.1.1. Effect of Cu Content

**Figure 2:**
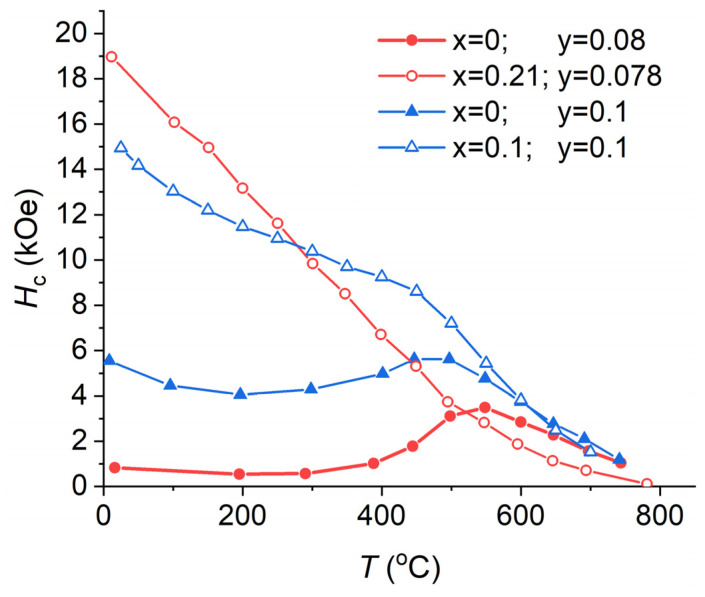
Temperature dependencies of the coercivity of the Sm(Co_0.978−*x*−*y*_Fe*_x_*Cu*_y_*Zr_0.022_)_7.3_ magnets, data from [17,36].

In [17], it was shown via X-ray and thermomagnetic analyses that in the Sm(Co_0.978−*y*_Cu*_y_*Zr_0.022_)_7.3_ (*y* = 0, 0.78, 0.10, 0.13 and 0.20) alloys without Fe after full heat treatment, with increasing Cu content, the volume fraction and the Curie temperature *T*_C_ of the 1:5 phase increases from 26 to 43% and decreases from 700 to 400 °C [39] (Table 1), respectively. Figure 1 shows the temperature dependencies of coercivity for the Sm(Co_0.978−*y*_Cu*_y_*Zr_0.022_)_7.3_ PM [17]. Apparently, for the entire range of Cu concentrations, the *H*_c_(*T*) dependencies exhibit a maximum at high temperatures. With the increasing Cu content, the maximum shifts to lower temperatures. This is caused by a decrease in the Curie temperature of the 1:5 phase, which mainly contains Cu (see Table 1). *H*_c_(*T*) changes similarly in alloys containing Fe (Figure 2). However, in the case of high Fe-containing PMs, the *H*_c_(*T*) dependence no longer has a maximum.

**Figure 3:**
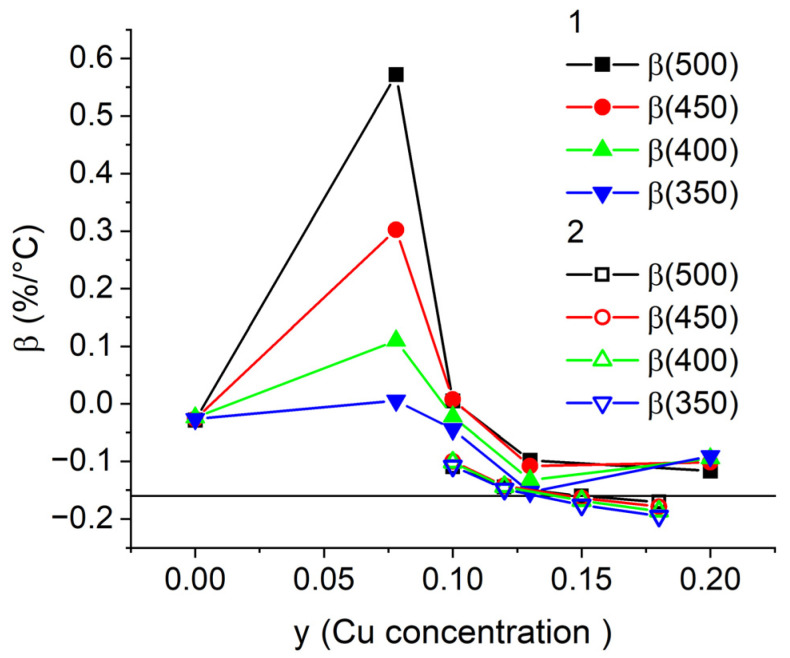
Dependence of temperature coefficients of coercivity β on Cu content in (1) the Sm(Co_0.978−*y*_Cu*_y_*Zr_0.022_)_7.3_ and (2) Sm(Co_0.88−*y*_Fe_0.1_Cu*_y_*Zr_0.02_)_7_ magnets.

Figure 3 shows the temperature coefficients of coercivity calculated from the data of refs [17,36] for temperature ranges starting from room temperature to *T* = 500 °C, 450 °C, 400 °C, and 350 °C for the Sm(Co_0.978−*y*_Cu*_y_*Zr_0.022_)_7.3_ and Sm(Co_0.88−*y*_Fe_0.1_Cu*_y_*Zr_0.02_)_7_ magnets. With the decrease in Cu content, the absolute value of the negative temperature coefficient of coercivity β decreases and then the β coefficient becomes positive. The maximum coefficient β achieved for compositions without Fe corresponds to the Cu content *y* = 0.08. However, this composition cannot be used in applications because the magnets have low *H*_c_ at room temperature (see Figure 1). To resist demagnetizing fields in the entire temperature range, the alloy must contain at least 5 wt. % Cu, or additional Fe have to be added (Figure 2). Comparison of β coefficients calculated for different ranges of operating temperatures shows that the most pronounced increase in the coefficient is observed for higher operating temperatures, i.e., 500 °C. Fe addition of *x* = 0.1 (Figure 2 and Figure 3, data from [36]) increases the absolute value of negative β by 0.05%/°C and decreases difference between coefficients of different temperature ranges. The latter is very convenient in terms of technology, because it is sufficient to estimate β in a narrow temperature range up to 350 °C. On the other hand, such behavior has to be taken into account when conducting research. Even small doping of Fe can complicate the study of the nature of magnetic hysteresis, because an increase in its content in the alloy decreases the volume fraction of the 1:5 phase [17,40]. Thus, numerous works state that it is impossible to measure the Curie temperature of the 1:5 phase [25]. Fe content *x* = 0.21 eliminates the abnormal temperature dependence (Figure 2). In this case, the β coefficient is −0.166 ± 0.002%/°C for all temperature ranges starting from room temperature up to 250–500 °C. Despite the fact that such a PM has a high operating temperature of more than 350 °C, the interpretation of its magnetic hysteresis is more consistent with the HEPM.

**Table 1:** Curie temperatures versus the content of elements in Sm-Co-Fe-Cu-Zr alloys.

*x*, *y*	*T*_C_(1:5), °C	*T*_C_(2:17), °C
Sm(Co_0.978−y_Cu_y_Zr_0.022_)_7.3_ [39]		
0	705	880
0.078	600	842
0.13	528	815
0.20	420	780
Sm(Co_0.88−*y*_Fe_0.1_Cu_y_Zr_0.02_)_7_ [36]		
0.1	550	850
0.12	510	850
0.15	360	850
0.18	280	850
Sm(Co_0.88−x_Fe_x_Cu_0.09_Zr_0.03_)_7_ [41]		
0	568	846
0.04	567	847
0.08	566	847
0.12	565	847

#### 3.1.2. Effect of Fe Content

Table 2 lists the compositions of Sm(Co_0.88−*x*_Fe*_x_*Cu_0.09_Zr_0.03_)_7_ magnets with Fe content *x* = 0–0.12 determined via chemical analysis and using APT.

**Figure 4:**
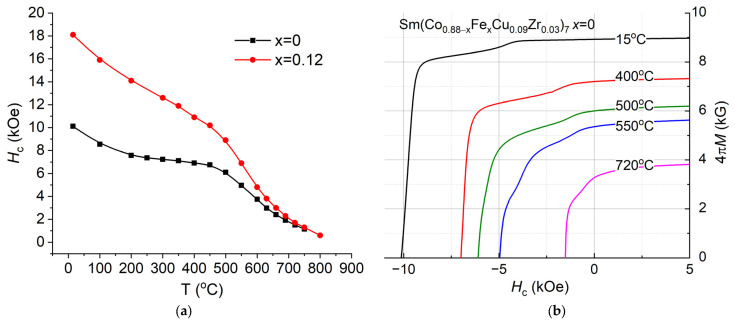
(**a**) Temperature dependencies of coercivity of Sm(Co_0.88−*x*_Fe*_x_*Cu_0.09_Zr_0.03_)_7_; (**b**) demagnetization curves of magnets with *x* = 0.

**Figure 5:**
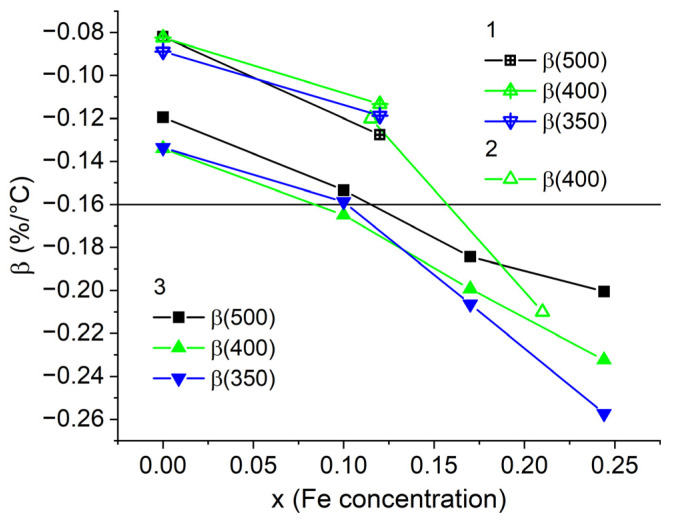
Dependence of temperature coefficients of coercivity β on Fe content in magnets: (1) Sm(Co_0.88−*x*_Fe*_x_*Cu_0.09_Zr_0.03_)_7_; (2) Sm(Co_0.92−*x*_Fe*_x_*Cu_0.06_Zr_0.02_)_7.6_; (3) Sm(Co_0.89−x_Fe*_x_*Cu_0.078_Zr_0.03_)_8.3_.

Figure 4a shows temperature dependencies of coercivity of the Sm(Co_0.88−*x*_Fe*_x_*Cu_0.09_Zr_0.03_)_7_ magnets with Fe content *x* = 0 and 0.12. As an example, Figure 4b demonstrates demagnetization curves which have been used to construct temperature dependencies of coercivity, as shown in Figure 4a for *x* = 0. With increase in the Fe content, the coercivity increases. As noted in [21,38], with increasing Fe content there is a decrease in the volume fraction of the 1:5 phase in the sample. The Fe addition slightly decreases the Curie temperatures of the 1:5 and 2:17 phases, as shown in Table 1. Figure 5 shows the dependencies of the β coefficient on the Fe content in the ranges from room temperature to *T* = 500 °C, 450 °C, 400 °C, and 350 °C, which are calculated from Figure 4a and data from [38,42]. The latter are characterized by a lower Sm content: Sm(Co_0.92−*x*_Fe*_x_*Cu_0.06_Zr_0.02_)_7.6_ and Sm(Co_0.89−*x*_Fe*_x_*Cu_0.08_Zr_0.03_)_8.3_, respectively. The horizontal line corresponds to β = −0.16%/°C, and data points below the line correspond to compositions without the abnormal temperature dependence of coercivity [38,42]. The figure demonstrates that with increasing Fe content, the absolute value of β increases. In addition, it is important to note that with an increase in Fe, before falling into contents without abnormal dependence (higher than *x* = 0.1 and 0.12 for Sm(Co,Fe,Cu,Zr)*_z_ z* = 8.3 and 7–7.6, respectively), the slope of β(*x*) dependencies changes. It is also interesting that in the range of compositions with abnormal dependence, a change in the Sm content shifts the dependencies β(*x*) parallel, which is convenient for choosing technological regimes. β coefficients calculated for different temperature ranges differ most strongly for compositions with a high Fe content, which do not exhibit abnormal dependence of coercivity.

#### 3.1.3. Effect of Sm Content

The effects of Sm content were studied in detail in [43] in the *z* concentration range from 6.7 to 9.1. It was shown that the smaller the value of z, the smaller the absolute value of the β coefficient due to the increased fraction of the 1:5 phase. Another reason for this effect can be that with decreasing volume fraction of the 1:5 phase, its Cu content increases [44].

**Figure 6:**
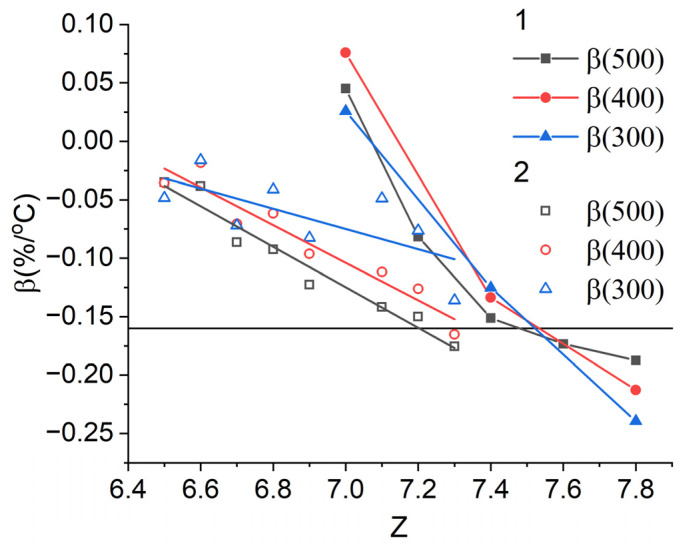
Dependence of the temperature coefficient of coercivity β on Sm content of the (1) Sm(Co_0.753_Fe_0.14_Cu_0.08_Zr_0.027_)*_z_* and (2) Sm(Co_0.795_Fe_0.09_Cu_0.09_Zr_0.025_)*_z_* magnets.

Figure 6 shows the dependencies of the temperature coefficient of coercivity β on the Sm content of the Sm(Co_0.753_Fe_0.14_Cu_0.08_Zr_0.027_)*_z_* and Sm(Co_0.795_Fe_0.09_Cu_0.09_Zr_0.025_)*_z_* magnets, which were calculated from the data [44] and [24] for two composition ranges with different Fe content. It can be seen that with the Fe content *x* = 0.09, the dependence is linear (the lines are an approximation using linear dependence), and the temperature dependencies of coercivity are abnormal [24]. With increasing Fe content *x* = 0.14, the slope of the β(*z*) dependence changes at *z* = 7.4. With decreasing the Sm content (higher *z*) at a higher Fe content, the decrease rate of the β coefficient is noticeably higher. The result is consistent with the results of Figure 6 and confirms the transition from abnormal to normal temperature dependence of coercivity at a higher Sm content with increasing Fe concentration.

#### 3.1.4. Effect of Zr Content

**Figure 7:**
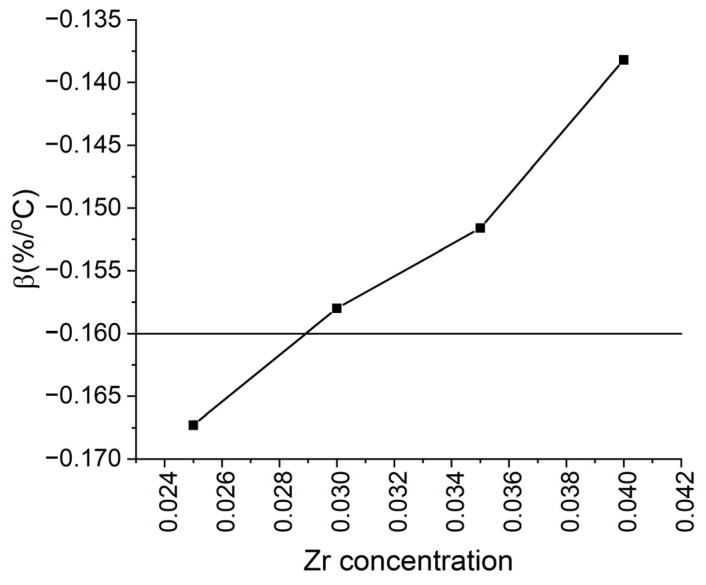
Dependence of the temperature coefficient of coercivity β on the Zr content of the Sm(Co_0.82−*v*_Fe_0.09_Cu_0.09_Zr*_v_*)_7.2_ magnets.

Zr plays the main role in the formation of the lamellar Z-phase, which stabilizes the cellular structure and increases its thermal stability. In addition, it is known that alloys with high Zr content are easier treated for solid solutions. On the other hand, its excessive amount forms Zr-Fe-Co inclusions, which remain within the grain boundaries and are parasitic in terms of magnetic hysteresis. In modern studies, the composition and crystal structure of the lamellar phase and its role in the formation of a cellular structure have been studied in detail [45,46]. However, studies on its effect on the temperature coefficient β of the HTPM are extremely limited [47]. Figure 7 shows the dependence of temperature coefficient β on the Zr content in the Sm(Co_0.82−*v*_Fe_0.09_Cu_0.09_Zr*_v_*)_7.2_ PM plotted from the data of [45]. With increasing Zr content in a composition typical of the HTPM, the absolute value of the β coefficient decreases. Unfortunately, despite the high quality of the microscopic work, the heat treatments in the work were carried out in a single mode, which—based on the poor squareness of the demagnetization curves and the absence of abnormal temperature dependence of coercivity in the compositions under consideration—turned out to be non-optimal. Thus, the coefficients were significantly lower than those in the works discussed above.

Based on the considered temperature dependencies of coercivity and the dependencies of the β coefficient on the element concentrations, the composition ranges of the HTPM with abnormal temperature dependence of coercivity can be estimated. Such magnets, if properly processed, can operate up to 550 °C. Thus, for Sm(Co_1−*x−y−v*_Fe*_x_*Cu*_y_*Zr*_v_*)*_z_*, *x* should vary in the range 0–0.14, *y* = 0.06–0.12, *v* = 0.022–0.03, and *z* = 6.9–7.4.

### 3.2. X-ray Diffraction and Atom Probe Tomography Study of HTPM

Despite numerous works devoted to the study of Sm-Co-Fe-Cu-Zr magnets using the APT method, the HTPM composition of Sm(Co_bal_Fe_0.10_Cu_0.10_Zr_0.03_)_7.2_ has been studied only recently [15]. However, the study of the temperature dependencies of coercivity has not been carried out; thus, it was not clear whether the high *H*_c_ was achieved at elevated temperatures. The Sm(Co_0.76_Fe_0.12_Cu_0.09_Zr_0.03_)_7_ composition studied in this work has been subjected to optimal heat treatment to obtain the high-coercivity state [41] and is characterized by a pronounced abnormal temperature dependence of coercivity, which is shown in Figure 4a.

According to the X-ray data, the sample contains 59 vol. % of the 2:17 phase with the lattice parameters *a* = 8.462 Å, *c* = 12.2188 Å, and 41 vol. % of the 1:5 phase. The 1:5 phase reflections are broadened and can be represented as a superposition of regions with the variable parameters *a* from 4.966 to 4.924 Å and *c* from 4.034 to 4.037 Å. Apparently, the 1:5 phase is stressed and has a continuous variation in the copper content from the interface with the 2:17 phase to the 1:5 center.

**Figure 8:**
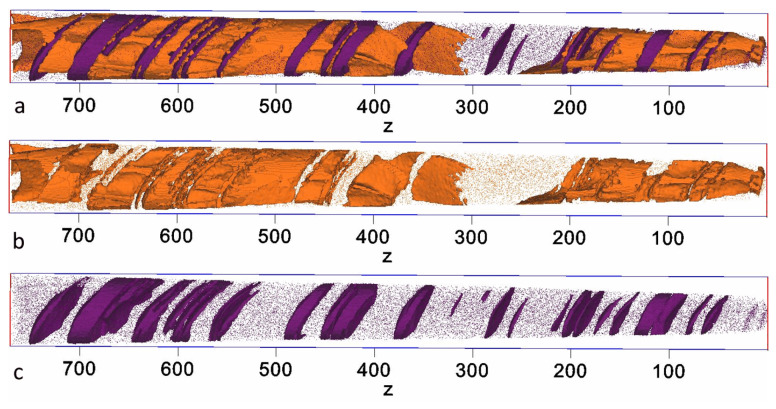
(**a**) Cu and Zr; (**b**) Cu; (**c**) Zr overlaid with the respective isoconcentration surfaces of 7 at.% Cu and 10 at.% Zr.

Figure 8 shows the 3D reconstruction of Cu and Zr distributions in the sample. Apparently, there is a considerable amount of the 1:5 phase in the sample. The 2:17 cell size is about 35–50 nm; that is considerably lower than the 100–150 nm characteristic of the HEPM cells [16,48]. On the other hand, the size of the boundary phase turns out to be much larger than the 5–8 nm characteristic of the HEPM boundaries.

**Figure 9:**
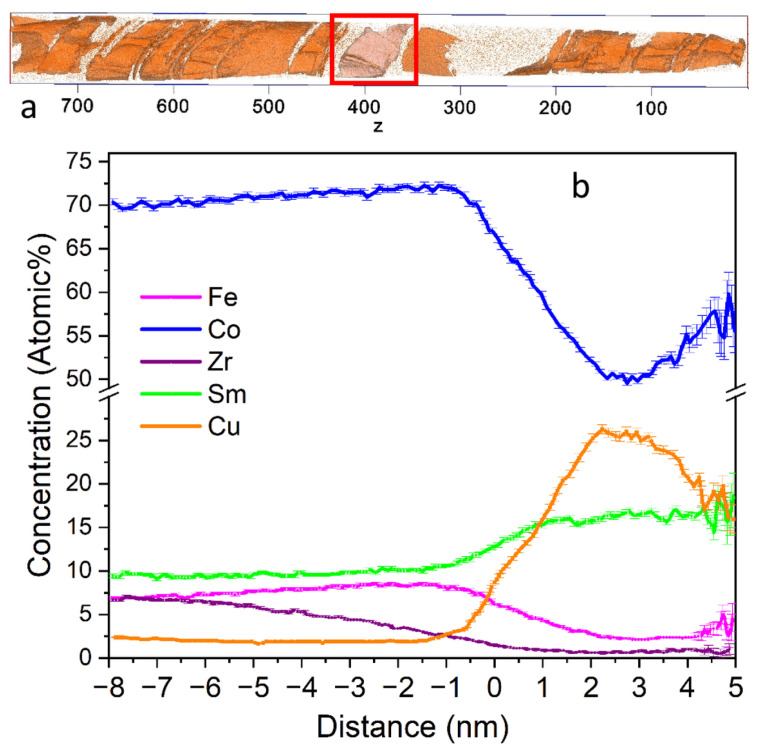
(**a**) Elemental distribution map of Cu with a 7 at.% isoconcentration surface (**b**); proxigram obtained from a representative Cu-rich region (marked with a rectangle in (**a**)) with a 0.1 nm bin width.

Figure 9b demonstrates a so-called proxigram, which was taken from the Cu-rich region (marked with a rectangle in Figure 9a, bin width = 0.1 nm). The vertical dashed line marks the beginning of the Cu-rich region, the right-hand limit represents the maximum Cu content. Interestingly, according to the proxigram, the Cu distribution does not have a single smooth maximum. Such a shape is evidence of the nonuniform thickness of the cell boundary phase. The thickness of the cell boundary phase is estimated to be about 10–12 nm, which is approximately two times higher than that of the HEPM.

**Figure 10:**
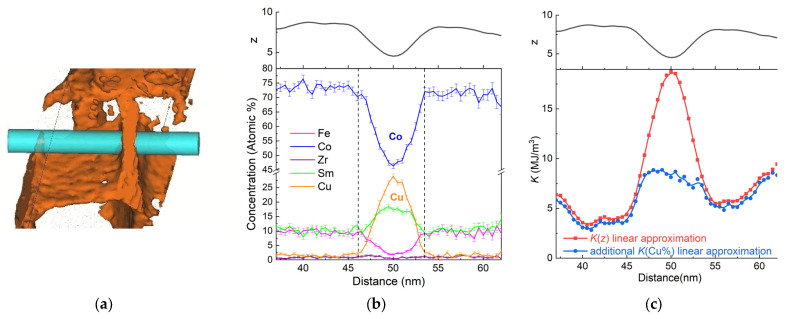
(**a**) Localization of the element distributions obtained using a 10-nm diameter cylindrical region of interest. (**b**) Element distributions and (**c**) anisotropy constant calculation *K*_1_.

Figure 10 shows element distributions in the cellular structure determined using APT. The region limited by dashed vertical lines corresponds to element distribution in the cell boundary region perpendicular to its surface. Outside this region, there is a cell interior phase. In the center of the cell boundary, there are minimums of Fe and Co distributions and maximums of Sm and Cu. The maximum Cu content reaches 26 at %. At the interfaces of the cell boundary and cell interior regions, there is a minimum Sm content and a maximum Fe content. The smoothed values of the stoichiometric *z* index of Sm(Co,Fe,Cu)_z_ were calculated from the APT element concentrations. The minimal *z* value corresponds to the center of the cell boundary phase and is approximately 4.8. *z* monotonously increases up to the interface with the cell interior, where it achieves a maximal value of ~9. In the cell interior phase, *z* is approximately 8. Such behavior of *z* suggests that, in the samples studied, the cell boundary phase is a region of monotonously changing stoichiometry from the maximum Sm_2_(Co,Fe,Cu)_17+δ_ at the interfaces to the minimum Sm(Co,Fe,Cu)_5−δ_ at the center. The appearance of *z* maximums at the borders of the boundary phase seems to be caused by the counter diffusion of Fe and Co into the 2:17 phase and Cu into the cell boundary phase in the course of the step cooling. In addition, the diffusion rate for different elements is different. In X-ray patterns [41], this causes the smearing of the peaks of the cell boundary phase.

Following the method suggested in [10], the anisotropy constant *K*_1_ was estimated from the APT data. From [49], it is known that in the range of *y* from 0.2 to 0.6, *K*_1_ changes almost linearly. Taking this dependence into account and suggesting a linear decrease in *K*_1_ with increasing *z* (from 1:5 to 2:17), the formula *K*_1_ = *K*_0_ + k_z_*z* + k_Cu%_*y*_Cu%_ can be derived, and the *K*_1_ distribution in Figure 10c is obtained. Here, the black curve is the experimental dependence of *z* on the distance in the sample (perpendicular to the boundary phase region). The red line characterizes the *K*_1_ estimate, taking into account the linear dependence of the anisotropy constant on *z*. The blue line additionally takes into account the decrease in *K*_1_ with the increasing Cu content. It can be seen from the resulting graph that, despite the boundary phase width of 12 nm, there are additional regions about 5-nm wide, with a reduced anisotropy coefficient on each side of the boundary. Thus, the *K*_1_ dependence is typical of the DW pinning at the interfaces of the boundary phase and the cell phase in the 2:17 phase (repulsive pinning).

Contrary to the results of [10], in which the *K*_1_ profile was determined for the samples of a high-energy composition (Sm lean or Fe rich) from the high-resolution transmission electron microscopy and energy-dispersive X-ray analysis, there is no minimum of *K*_1_ in the center of the boundary phase. Regions of the lower anisotropy constant are caused not only by the Cu diffusion, but also by the counter diffusion of Fe and Co, and are characterized by a local maxima of *z*. In [50], it has been shown that minimal replacement of Co by Cu and Fe in YCo_5_ increases the anisotropy constant. In the case of SmCo_5_, *K*_1_ dependencies on low Fe and Cu content can qualitatively be the same. Thus, a finer account of substituting elements can change the *K*_1_ dependence obtained in the boundary phase of the high-temperature PM.

## 4. Conclusions

To determine the range of compositions of Sm-Co-Fe-Cu-Zr alloys most suitable for high-temperature PMs, the dependencies of the temperature coefficient of the coercivity β on the content of elements are analyzed. It has been established that PMs with operating temperatures up to 550 °C can be obtained with optimal processing of Sm(Co_1*−x−y−v*_Fe*_x_*Cu*_y_*Zr*_v_*)*_z_* alloys, where *x* can vary in the range from 0 to 0.14, *y* from 0.06 to 0.12, *v* from 0.022 to 0.03, and *z* from 6.9 to 7.4. In this case, the absolute value of β and room temperature coercivity do not exceed 0.16%/°C and 20 kOe, respectively.

To elucidate the nature of abnormal temperature dependence of coercivity, a quantitative study using atom probe tomography was carried out for the Sm(Co_0.76_Fe_0.12_Cu_0.09_Zr_0.03_)_7_ sample, which has the best high-temperature properties. It was found that the HTPM has a 2–3-times-smaller cell size for the 2:17 phase. The average width of the boundary phase in the HTPM is about 12 nm, in contrast to previously published data on HEPMs of 5–8 nm. The boundary phase is an area of monotonic change in stoichiometry from Sm(Co,Fe,Cu)_5−δ_ to Sm_2_(Co,Fe,Cu)_17+δ_. Thus, current and future studies should be aimed at determining the optimal values of the cell size; the width of the boundary phase; as well as the influence of their shape, spatial ordering, and nanostructure on the main magnetic characteristics of PMs of this type.

Based on the element concentrations, the profile of the anisotropy constant is determined in the boundary phase of the cellular structure. It has been established that interface regions with a lower anisotropy constant adjoin the boundary phase, which in combination with other mechanisms, provide a pinning of domain walls in high-temperature PMs with abnormal temperature dependence. Therefore, future research should focus on the determination of the optimal ratio between the sizes of regions with reduced anisotropy and the thickness of the domain wall, as well as the optimal anisotropy constant profile in the high-temperature PMs with abnormal temperature dependence of coercivity for further advancement of operating temperatures.

## Figures and Tables

**Table 2:** Composition of Sm(Co_0.88−*x*_Fe*_x_*Cu_0.09_Zr_0.03_)_7_ magnets in at. % determined via chemical analysis and during APT.

Elements	Sm	Co	Fe	Cu	Zr	Total
*x* = 0, chemical	12.5	77.0	0.0	7.9	2.6	100
*x* = 0, APT	12.8	73.4	0.1	8.7	2.1	97.1
*x* = 0.04, chemical	12.5	73.5	3.5	7.9	2.6	100
*x* = 0.08, chemical	12.5	70.0	7.0	7.9	2.6	100
*x* = 0.12, chemical	12.5	66.5	10.5	7.9	2.6	100
*x* = 0.12, APT	11.7	68.5	5.9	6.9	2.5	95.7

## Data Availability

Data is available upon request.
